# Effectiveness of a Controlled 5-FU Delivery Based on FZD10 Antibody-Conjugated Liposomes in Colorectal Cancer In vitro Models

**DOI:** 10.3390/pharmaceutics12070650

**Published:** 2020-07-10

**Authors:** Maria Principia Scavo, Annalisa Cutrignelli, Nicoletta Depalo, Elisabetta Fanizza, Valentino Laquintana, Giampietro Gasparini, Gianluigi Giannelli, Nunzio Denora

**Affiliations:** 1 Personalized Medicine Laboratory, National Institute of Gastroenterology “S. deBellis”, Via Turi 26 Castellana Grotte, 70125 Bari, Italy; 2 Department of Pharmacy-Drug Science, University of Bari, Via E. Orabona 4, 70125 Bari, Italy; annalisa.cutrignelli@uniba.it (A.C.); valentino.laquintana@uniba.it (V.L.); nunzio.denora@uniba.it (N.D.); 3 Institute for Chemical and Physical Processes (IPCF)-CNR SS Bari, Via Orabona 4, 70125 Bari, Italy; n.depalo@ba.ipcf.cnr.it (N.D.); e.fanizza@ba.ipcf.cnr.it (E.F.); 4 Department of Chemistry, University of Bari, Via E. Orabona 4, 70125 Bari, Italy; 5 Oncology Unit, Hospital San Filippo Neri, 00135 Rome, Italy; giampietro.gasparini@hotmail.it; 6 Scientific Direction, National Institute of Gastroenterology “de Bellis”, Via Turi 26 Castellana Grotte, 70125 Bari, Italy; gianluigi.giannelli@irccsdebellis.it

**Keywords:** liposomes, target delivery nanosystem, FZD10 protein, colon cancer therapy

## Abstract

The use of controlled delivery therapy in colorectal cancer (CRC) reduces toxicity and side effects. Recently, we have suggested that the Frizzled 10 (FZD10) protein, a cell surface receptor belonging to the FZD protein family that is overexpressed in CRC cells, is a novel candidate for targeting and treatment of CRC. Here, the anticancer effect of novel immuno-liposomes loaded with 5-Fluorouracil (5-FU), decorated with an antibody against FZD10 (anti-FZD10/5-FU/LPs), was evaluated in vitro on two different CRC cell lines, namely metastatic CoLo-205 and nonmetastatic CaCo-2 cells, that were found to overexpress FZD10. The anti-FZD10/5-FU/LPs obtained were extensively characterized and their preclinical therapeutic efficacy was evaluated with the MTS cell proliferation assay based on reduction of tetrazolium compound, scratch test, Field Emission Scanning Electron Microscopes (FE-SEM) investigation and immunofluorescence analysis. The results highlighted that the cytotoxic activity of 5-FU was enhanced when encapsulated in the anti-FZD10 /5-FU/LPs at the lowest tested concentrations, as compared to the free 5-FU counterparts. The immuno-liposomes proposed herein possess a great potential for selective treatment of CRC because, in future clinical applications, they can be encapsulated in gastro-resistant capsules or suppositories for oral or rectal delivery, thereby successfully reaching the intestinal tract in a minimally invasive manner.

## 1. Introduction

Colorectal cancer (CRC) is the second leading cause of cancer death worldwide [1]. Cytotoxic drugs-based chemotherapy is the main treatment in cancer management, in addition to surgery, radiation, and biological therapies [2]. 5-Fluorouracil (5-FU) is widely used in CRC chemotherapy, as also other different drugs employed to interfere with cell replication by acting as nucleoside analogs and leading to S-phase arrest, or by damaging deoxyribonucleic acid (DNA) [3]. Unfortunately, owing to the short half-life (5–14 min), poor membrane permeability and rapid metabolism in the body, continuous administration of high doses of 5-FU is required to maintain the minimum therapeutic serum concentration; this is usually associated with numerous side effects and severe toxicity [4]. Nanostructured carriers have been proposed as an alternative therapeutic approach, since they can be properly surface-functionalized and consequently reach a specific target on pathological cells. Therefore, targeted drug delivery nanosystems can ensure the specific release of drugs in tumor areas, at lower and more efficient doses than conventional treatments, thus maximizing the bioavailability of anticancer agents and their uptake into the cancer tissue, while reducing side effects and drug distribution to healthy tissues. Drug-loaded nanovectors have also been demonstrated to be able to overcome the canonical drug-resistance pathways, e.g., membrane pumps, for cellular internalization that usually occurs when using small therapeutic molecules [5,6]. Furthermore, drug delivery nanocarriers can be protected by using pH-dependent polymer coatings or pH-dependent encapsulation systems, to prevent their degradation in the gastric tract [7,8].

Liposomes (LPs) are lipid-based nanostructures with a hydrophilic core and a lipophilic shell, where different types of drugs can be encapsulated [9,10]. The small size of LPs is fundamental to promote extravasation and accumulation phenomena in tumor sites. In the literature, several examples of liposomes have been proposed for CRC treatment, by intravenous, oral or rectal administration [11,12]. The coupling of the LPs surface with selected antibodies, as ligands able to recognize and bind specific plasma membrane proteins that are overexpressed in cancer tissues, allows immuno-LPs to be created, taking a further step forward in the development of efficient targeted drug delivery nanosystems [13,14].

The FZD proteins (FZDs) are the main cell surface receptors for the WNT family of ligands and include ten members (FZD1–FZD10). FZDs are involved in the regulation of several cell processes, which occur not only in normal development of different body systems, but also in various cancers. Indeed, numerous studies proved that FZDs play a crucial role in different cancer functions, thus promoting proliferation, migration, invasion, angiogenesis, and also chemoresistance. Furthermore, modulation of expression levels for specific FZD depending on up- or downregulation of the corresponding messenger RNA (mRNA) was observed in different cancers tissues [15,16,17,18,19,20]. Among the FZD proteins, FZD10 was suggested to be one of the most promising receptors for the development of targeted therapy of CRC. Indeed, a study by S. Nagayama et al. evaluated the immunohistochemical expression patterns of FZD-10 in tissue samples of patients affected by CRCs. Interestingly, their study indicated that FZD10 was expressed only in cancer cells and it was absent in adjacent normal cells [21,22]. Furthermore, we have reported a study performed on tissues of three different cancers, namely CRC, melanoma and gastric cancer, demonstrating a strong correlation between the expression levels of FZD10 and different tumor stages. In the colon, a significant increase of FZD10 expression in membrane and cytoplasm and, concomitantly, a significant reduction in nuclei expression were observed, passing from nondysplastic to malignant tissue [22,23]. Based on these premises, FZD10 can be effectively exploited as a novel candidate target with a good potential in the development of selective therapeutic approaches for CRC.

The aim of the study will be to create stealth LPs functionalized with FZD 10 antibody and loaded with 5-FU (anti-FZD10/5-FU/LPs), towards the development of efficacious nanostructured formulations for the selective delivery and sustained release of anticancer drug to CRC sites by exploiting active targeting. A deep in vitro investigation on their preclinical therapeutic efficacy in CRC treatment will be performed.

## 2. Material and Methods

5-fluorouracil (5-FU, MW = 130.077 g/mol) was purchased from Sigma-Aldrich (Milan, Italy) and used as stock solution in ethanol (380 mM). The Liposomes Kit, composed of cholesterol (Chol, 9 µmol/package), L-α-Phosphatidylcholine (egg yolk, 63 μmol/package) and stearylamine (18 μmol/package), N-(3-dimethylamino-propyl)-N’-ethylcarbodiimide hydrochloride (EDC), N-hydroxysulfosuccinimide (sulfo-NHS) and phosphotungstic acid (99.995%) were purchased from Sigma-Aldrich (Milan, Italy). 1,2-stearoyl-*sn*-glycero-3-phosphoethanolamine-N-[carboxy(poly(ethylene glycol)-2000)] (DSPE-PEG2000-COOH) and 1,2-distearoyl-*sn*-glycero-3-phosphoethanolamine-N-[methoxy(polyethylene glycerol)-2000] (DSPE-PEG2000) were purchased from Avanti Polar Lipids (Alabaster, AL, USA). Fetal bovine serum, penicillin/streptomycin and glutamine were purchased from Thermo-Fisher Scientific (Waltham, MA, USA). Polyclonal antibody against FZD10 was purchased from ABCAM (Cambridge, UK). Mouse monoclonal anti-human vimentin and rabbit polyclonal anti-human phospho-Paxillin were purchased from Cell Signaling Technology (Beverly, MA, USA) Goat anti-Mouse IgG (H + L), Superclonal™ Recombinant Secondary Antibody, Alexa Fluor 488 and goat anti-Rabbit IgG (H + L) Cross-Adsorbed Secondary Antibody, Alexa Fluor 555 were purchased from Thermo Fisher Scientific (Waltham, MA, USA) Prolong gold antifade reagent containing the nuclear staining 4′,6-diamidino-2-phenylindole dichloride (DAPI) was purchased from Invitrogen (Carlsbad, CA, USA). CellTiter 96 AQueous One Solution Cell Proliferation Assay (MTS) was purchased from Promega (Madison, WI, USA). All aqueous solutions were prepared using water obtained from a Milli-Q Gradient A-10 system (Millipore, 18.2 MΩcm, organic carbon content ≥ 4 µg/L). All other solvents and reagents were of analytical grade.

### 2.1. Preparation of Liposomes

Nude LPs loaded with 5-FU (5-FU/LPs) were prepared using the ether injection method [24]. Briefly, 2.738 mL of an ether/chloroform (1:1, *v*/*v*) solution, 0.274 mL of a chloroform solution of lipid mix containing cholesterol, phosphatidylcholine and stearylamine (7:3:1 molar ratio) and 11 µL of a chloroform solution of DSPE-PEG2000 were mixed in a glass vial using a magnetic stirrer, brought to a lipid mix/DSPE-PEG2000 molar ratio of 3:0.3. Then, 3 mL of aqueous solution 5-FU (38 mM) were rapidly injected into the organic lipid solution using a sterile glass syringe. LPs were then obtained according to the previously reported experimental procedure [24]. Empty LPs were prepared following the same protocol, but without 5-FU addition.

For the preparation of 5-FU/LPs conjugated with FZD10 antibody (anti-FZD10/5-FU/LPs), 5-FU/LPs were previously surface-functionalized with carboxylic groups (5-FU/LPs-COOH). For this purpose, the above-described experimental protocol was followed, using as starting lipid mixture, 2.738 mL of an ether/chloroform (1:1, *v*/*v*) solution, 0.274 mL of a chloroform solution of lipid mix, 3 µL of a chloroform solution of DSPE-PEG2000 and 8 µL of a chloroform solution of DSPE-PEG2000-COOH. The final total lipid concentration in all the liposomal formulations was kept constant at 3 mM. After the purification procedure, 5-FU/LPs-COOH (300 µL) dispersed in ultrapure distilled water were activated by adding sulfo-NHS (11 mg) and EDC (9 mg). The reaction mixture was left under gentle stirring overnight at room temperature. Then, the samples were ultracentrifuged at 10,000× *g* for 40 min at 4 °C to remove excess crosslinking reagents. The activated 5-FU/LPs-COOH were recovered as pellets, dispersed in 300 µL of PBS and incubated with 5 µg of anti-FZD10 antibody. The mixture was gently stirred overnight at room temperature. Finally, the anti-FZD10/5-FU/LPs were purified by ultracentrifugation at 10,000× *g* at 4 °C for 40 min to remove unbound antibody. All the liposomal formulations were lyophilized (Christal freeze dryer alpha 1-4 LSC) and then reconstituted in PBS or water prior to their use or characterization. The experimental details on the indirect detection of FZD10-antibody bound onto the surface of LPs are reported in Supplementary Materials.

### 2.2. Evaluation of Encapsulation Efficiency (EE%)

The encapsulation efficiency (EE%) of 5-FU encapsulated in 5-FU/LPs or anti-FZD10/5-FU/LPs was evaluated according to the following formula:

EE% = Wt/Wi × 100(1)

where Wt is the amount of drug effectively incorporated into the liposomal formulation and Wi the total quantity of 5-FU initially added during the preparation. To evaluate the drug content, samples were lyophilized and treated with methanol (1:100 dilution) and then the absorbance spectra (Perkin Elmer Double beam UV-Visible Spectrophotometer Lambda Bio 20) were recorded at 265 nm versus a methanol solution containing the same lipid mixture used for liposome preparation (baseline) [20]. Three measurements were performed on three different batches for each liposomes formulation. A calibration curve of 5-FU was generated by measuring the drug absorbance at 265 nm of standard methanol solutions at concentrations ranging from 0.25 mM to 0.05 mM. 

### 2.3. In vitro Drug Release Study

1 mL of FZD10-anti/5-FU/LPs was introduced into dialysis tubing (cut-off 3.5 kDa, Spectrapore) and dialyzed against 50 mL of PBS (10 mM and pH=7.4, outer medium). The dialysis was conducted at 37 °C in a water bath shaker. At defined time intervals, over 48 h, 100 µL of outer medium were collected, removed and replaced with fresh PBS. Each collected aliquot was lyophilized and solubilized in methanol; the drug concentration was determined by measuring the UV-Vis absorbance at 265 nm. The calibration curve described in the previous paragraph was used for the quantitative evaluation of the released drug. Measurements were conducted three times per sample.

### 2.4. Cell Culture

The CaCo-2 cell line is originally derived from a primary colon adenocarcinoma (Cancer Coli-2) and was established by Jorgen Fogh at the Sloan Kettering Cancer Research Institute, from a Caucasian male (ATCC). The CoLo-205 cell line has been established from ascites fluid obtained from a male patient with metastatic adenocarcinoma of the colon (ATCC). CaCo-2 cells were cultured in Eagle’s Minimum Essential Medium, (GibCo), which was added with 10% of fetal bovine serum, 1% of penicillin/streptomycin and 1% of glutamine. For the CoLo-205 cell line, ATCC-formulated Roswell Park Memorial Institute (RPMI)-1640 Medium was employed, with the addition of 10% of fetal bovine serum (10%), 1% of penicillin/streptomycin (1%) and 1% of glutamine (1%). When the cell lines confluence was about 70%, the cell layer was rinsed with PBS, and trypsinized, for the subsequent in vitro experiments.

### 2.5. Cells Proliferation Assay

The MTS cell proliferation assay (CellTiter 96^®^ AQueous One Solution Cell Proliferation Assay, Promega) was used to determine metabolic activity in CaCo-2, CoLo-205 cell lines. Briefly, cells were seeded into 96-well plates at a density of 2 × 10^3^ cells/well. After 24 h, the cells were treated with free drug (5-FU), empty LPs, 5-FU/LPs and anti-FZD10/5-FU/LPs, at 5-FU concentrations ranging from 1 µmol to 10 µmol (5-50 µM, in terms of total lipid concentration for the liposomal formulations), for 24, 48 or 72 h. After cell incubation with the different samples, cells were treated with the MTS tetrazolium compound for three additional hours and the absorbance was measured at a wavelength of 490 nm using a Perkin Elmer Victor Plate Reader (Mechelen, Belgium). 

### 2.6. In vitro Investigation by Field Emission Scanning Electron Microscopy

CaCo-2 and CoLo-205 cells were seeded on silicon wafers (Ted Pella Inc., Redding, CA, USA) at a density of 2 × 10^3^/well in 24-wells plates and at a subconfluent density, exposed to 5-FU/LPs and anti-FZD10/5-FU/LPs at a drug concentration of 2 µM for 6, 24 and 96 h. After cell washing with PBS, all cell lines were fixed with 3% glutaraldehyde in PBS for 1 h at 4 °C and incubated in 1% OsO_4_ for 1 h. The samples were washed five times with aqueous solution of sodium cacodylate (0.005 M, pH 7.2). Then, sequential cell treatment steps with water/acetone mixtures, gradually increasing the acetone volume from 20% to 100%, were carried out to accomplish the complete dehydration process of the cells. The same fixation procedure was carried out after deposition on silicon chips also for the untreated cell lines. The fixed and dried cells were coated with a uniform Au metal film, a few nanometers thick, that was deposited on the samples placed on silicon chips using a turbomolecular pump SC7620 Mini Sputter/Glow Discharge System, Quorum Technologies, and imaged using a Zeiss Sigma FE-SEM. A constant Extra-High Tension (EHT) value of 3.00 kV and a working distance (WD) ranging from 1.8 to 3 mm were set for the FE-SEM observation. For each experiment and sample, a representative FE-SEM micrograph was selected after the collection of a set of images resulting from three replications of the same experiment [20]. 

### 2.7. In vitro Scratch Assay

CaCo-2 and CoLo-205 cells were plated in six-well plates to create a cell monolayer, following the protocol described by C. Liang et al [25]. Briefly, a “scratch” with a p200 pipette tip was created on the cell monolayer, for each sample and experiment. After removal of the debris by washing with 1 mL of culture medium, an ultrafine tip marker was used to create markings on the outer bottom of the plate that represent reference points close to the scratch. For the scratch assay, each cell line was incubated with 3 mL of medium containing free 5-FU, 5-FU/LPs or anti-FZD10/5-FU/LPs at a 5-FU concentration of 2 µmol for 24 or 48 h. The experiments were conducted at 37 °C. Untreated cells were used as control for each tested cell line. At 0, 24 and 48 h, the plates were placed under a confocal microscope Nikon Eclipse Ti2, for phase-contrast assessment to acquire the images of the scratch before and after cell incubation with the different samples. 

### 2.8. Immunofluorescence Analysis

CaCo-2 and CoLo-205 cells were seeded into four-well slides chambers at a density of 10 × 10^3^ cells per well at 37 °C and treated with free 5-FU, 5-FU/LPs or anti-FZD10/5-FU/LPs, respectively, for 24 or 48 h. Untreated cells were used as control. Subsequently, treated and untreated cells were washed three times with PBS, fixed with cold ethanol (96°) for 25 min and permeabilized with an aqueous solution of Triton X-100 (0.5%) in PBS for 15 min. Then cells were blocked with an aqueous solution of normal serum (5%) in PBS for 1 h and incubated at 4 °C over-night with the two primary antibodies mix. The protocol applied has been previously described [19], with the following antibodies: mouse monoclonal anti human Vimentin (diluted 1:250 in Blocking) and rabbit polyclonal anti human phospho-Paxillin (Tyr118) (diluted 1:200 in Blocking). Then, cells were washed twice with PBS and incubated with a specific green-fluorescent (goat anti-mouse IgG (H + L) secondary antibody Alexa Fluor 488 conjugate) and red-fluorescent (goat anti-rabbit IgG (H + L) superclonal secondary antibody, Alexa Fluor 555) secondary antibodies for 1 h, at room temperature, in the dark. After washing with PBS, the cells were mounted using prolonged gold antifade reagent containing DAPI (blue). Images were acquired using the Nikon confocal microscope Eclipse Ti2 and the fluorescence intensity was quantified using Image J software (number of pixels/area). Five different areas from the single well for each single independent experiment performed in triplicate were randomly selected [26].

### 2.9. Dynamic Light Scattering Analysis and ζ-Potential Investigation

The hydrodynamic diameter (size), the size distribution and the colloidal stability of the liposomal formulations were detected using a Zetasizer Nano ZS, Malvern Instruments Ltd., Worcestershire, UK (DTS 5.00), as previously reported [20]. Three measurements were performed on at least three different batches of each vesicles formulation, and results are reported together with the corresponding standard deviation.

### 2.10. Morphological Characterization by Transmission Electron Microscopy (TEM)

For TEM investigation, liposomal formulations deposition was made onto 400 mesh amorphous carbon-coated Cu grids. For each sample, 5 µL of aqueous dispersion of LPs were dropped on the grid and the solvent was left to evaporate. Then, the grid was placed in contact with the top surface of a drop composed of an aqueous phosphotungstic acid solution (2%, *v*/*v*) for 30 s. Finally, ultrapure free water was used to wash the grid. TEM measurements were carried out using a Jeol JEM-1011 microscope, working at an accelerating voltage of 100 kV and fitted with an Olympus Quemesa Camera (11 Mpx).

### 2.11. Statistical Analysis

All the experiments were performed three times and all results are presented as mean ± standard deviation. Data were analyzed by one-way ANOVA followed by Bonferroni test using GraphPad Prism version 5 for Windows (GraphPad Software, San Diego, CA, USA) and statistical significance was set at *p* < 0.001.

## 3. Results

### 3.1. Liposomes Formulation and Characterization

**Figure 1:**
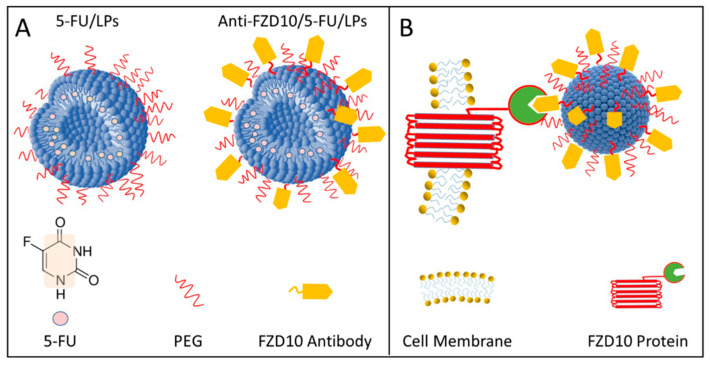
Pictorial sketch of PEG (Poly ethylene glycol) stabilized and 5-Fluorouracil (5-FU)-loaded liposomes (LPs) before (5-FU/LPs) and after (anti-FZD10/5-FU/LPs) conjugation with FZD10 antibody, according to the corresponding legend (**A**). Schematic representation of molecular recognition between anti-FZD10/5-FU/LPs and FZD10 protein expressed on the colorectal cancer (CRC) cell membrane surface. Drawings not to scale. (**B**).

Poly ethylene glycol (PEG)-stabilized and 5-FU-loaded LPs (5-FU/LPs) were decorated with anti-FZD10 and in vitro tested to evaluate their effectiveness for selective CRC treatment (Figure 1A). A schematic illustration of active targeting of CRC by molecular recognition between anti-FZD10/5-FU/LPs and FZD10, expressed at the plasma membrane surface of CRC cells, is shown in Figure 1B.

For the preparation of anti-FZD10/5-FU/LPs, 5-FU/LPs bearing carboxylic groups were covalently conjugated with the primary amine groups of anti-FZD10 antibody by crosslinking chemistry. The effective occurrence of the conjugation reaction between FZD10-antibody and 5-FU/LPs was assessed by labeling the surface of anti-FZD10/5-FU/LPs with a specific secondary antibody-Alexa Fluor 555. The indirect detection of FZD10-antibody on anti-FZD10/5-FU/LPs was demonstrated by UV-Vis absorbance spectroscopy analysis, as described in the Supplementary Materials. In particular, the presence of a peak centered at 555 nm, due to the covalent binding of the dye conjugated secondary antibody to the surface of anti-FZD10/5-FU/LPs, can be observed in the absorption spectrum of anti-FZD10/5-FU/LPs, after their incubation with labelled secondary antibody (Figure S1B, Supplementary Materials), while the same peak does not appear in the absorbance spectrum of untreated anti-FZD10/5-FU/LPs (Figure S1A, Supplementary Materials).

The formulated LPs, namely 5-FU/LPs and anti-FZD10/5-FU/LPs, were characterized in terms of size, morphology and colloidal stability by performing DLS investigation, TEM analysis and ζ-potential measurements (Table 1). DLS investigation revealed that the mean hydrodynamic diameter of bare 5-FU/LPs was equal to (155 ± 47) nm, and this domain size was a consequence of the porosity of the polycarbonate membrane used for the extrusion. Anti-FZD10/5-FU/LPs exhibited a larger average size (193 ± 12) nm, as expected when the surface functionalization of liposomes is carried out. The values of polydispersion index (PDI) denoted the presence of a fairly uniform LPs population in dimensional terms for both the formulations (Table 1).

**Figure 2:**
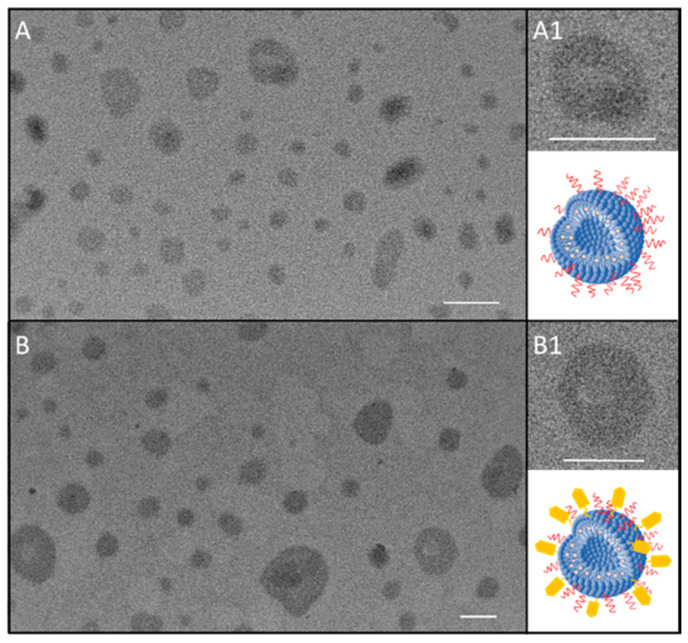
Representative transmission electron microscopy (TEM) micrograph of 5-FU/LPs (**A**,**A1**) and anti-FZD10/5-FU/LPs (**B**,**B1**). High magnification close-up of 5-FU/LPs (**A1**) and anti-FZD10/5-FU/LPs (**B1**) along with the corresponding sketch. Scale bar 100 nm.

The representative TEM micrographs of 5-FU/LPs (Figure 2A,A1) and FZD10-anti/5-FU/LPs (Figure 2B,B1) proved the formation of nanostructures with quite round shape for both the liposomal formulations. In the TEM micrographs of 5-FU/LPs (Figure 2A,A1) and anti-FZD10/5-FU/LPs (Figure 2B,B1), nanostructures with a rounded shape can be observed for both the formulated LPs, having size ranging from 30 to 110 nm and from 40 to 195 nm for 5-FU/LPs and FZD10-anti/5-FU/LPs, respectively. Furthermore, for both the bare and the engineered LPs, some nano-objects revealing a darker, circular bilayer and a brighter interior space, as typically ascribed to LP structures, can be appreciated, as highlighted in the two high magnification close-ups (Figure 2A1,B1).

The TEM observations resulted in a good agreement with the data obtained by DLS investigation, also considering that the deposition procedure of samples on the TEM grid, performed before TEM analysis, induced a drying process and consequently a shrinking of the soft organic matter based LPs.

The ζ-potential measurements highlighted the presence of an overall negative charge at the surface of both investigated formulations, as the phosphate moieties of the phospholipids used for the preparation of 5-FU/LPs and anti-FZD10/5-FU/LPs are expected to be exposed onto their surface (Table 1). 

The decrease in drug EE% recorded for anti-FZD10/5-FU/LPs can be reasonably explained by taking into account the different steps required for the conjugation reaction, starting from bare 5-FU/LPs: the first incubation with the crosslinking agents, the subsequent centrifugation to remove the excess of reagents, the second incubation with the FZD10 antibody and the final centrifugation to remove the unbound antibody molecules.

The drug release study for FZD10-anti/5-FU/LPs, monitored by UV-Vis absorption spectroscopy, is reported in Figure S2 of Supplementary Materials.

### 3.2. Effectiveness of Formulated Liposomes on Cell Viability

**Figure 3:**
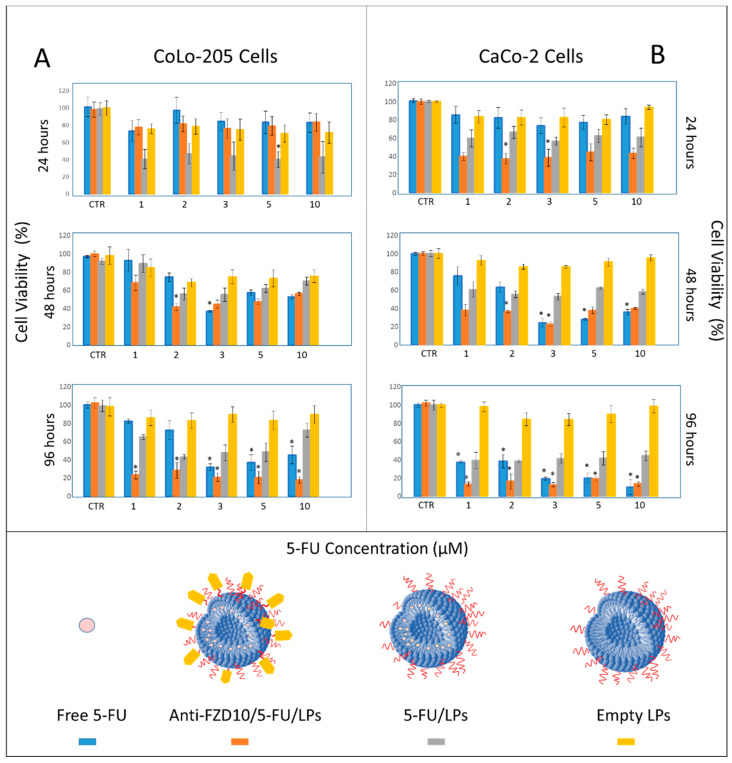
Cell viability, evaluated by MTS cell proliferation assay, of CoLo-205 (**A**) and CaCo-2 (**B**) cells after incubation with free 5-FU, 5-FU/LPs, anti-FZD10/5-FU/LPs and empty LPs at 5-FU concentrations ranging from 1 to 10 µM (from 5 to 50 µM in terms of total lipid concentration) for 24, 48 and 96 h. For each cell line, control was untreated cells. The experiments were conducted in triplicate. (*) *p* < 0.001 versus control.

CoLo-205 and CaCo-2 cells were selected as FZD10-positive CRC cell lines (Figure S3, Supplementary Materials). The effect of 5-FU and anti-FZD10/LPs on CoLo-205 and CaCo-2 cells viability was evaluated by MTS proliferation assay. For comparison, free 5-FU and nontargeted LPs were tested. The effect of the empty LPs (having a ζ-potential value of (−32.84 ± 3.42) mV) on cell viability was also assessed, in order to investigate on the cytotoxicity of liposomal vectors. In particular, the two cell lines were treated with free 5-FU, 5-FU/LPs and anti-FZD10/5-FU/LPs at drug concentrations ranging from 1 to 10 µM (in the range from 5 to 50 µM, in terms of total lipid concentration for the liposomal formulations), at 24, 48, and 96 h. CoLo-205 and CaCo-2 cell viability is reported in Section 3.3. The viability of both colon cancer cell lines was always greater than 80% after their incubation with empty LPs, thus indicating very low toxicity in the lipid concentration range, at each tested time incubation (Figure 3, yellow bars). For CoLo-205 cells, at the lowest tested drug concentration values, namely 1 and 2 µM, the cell viability was only minimally reduced (always higher than 75%) within 96 h, when cells were incubated with free 5-FU (Figure 3A, blue bars). As compared to free 5-FU, the nontargeted LPs were found to exhibit an enhanced cytotoxic activity, for each tested time incubation, at a drug concentration of 2 µM (Figure 3, grey bars). Conversely, in the range between 3 and 10 µM, the effectiveness of free 5-FU in affecting the cell viability was time-dependent (Figure 3A, blue bars); the bare 5-FU/LPs induced a higher cytotoxic effect than free 5-FU by reducing the cell viability up to about 40% only within the first 24 h (Figure 3A, grey bars). In the case of anti-FZD10/5-FU/LPs, the cell-killing effects were always time-dependent from 24 to 96 h, for each tested drug concentration (Figure 3A, orange bars). The cell viability was always lower than 30% when the cells were treated with anti-FZD10/5-FU/LPs for 96 h, in the entire tested range of drug concentrations (Figure 3A, orange bars). At a 5-FU concentration of 2 µM, the use of anti-FZD10/5-FU/LPs significantly (*p* < 0.001 versus control) reduced the cell viability up to (42 ± 3) and (28 ± 8)% at 48 and 96 h, while in the case of free 5-FU, only up to (74 ± 4) and (73 ± 10)%, thus resulting not statistically significant (Figure 3A, orange and blue bars). The cell viability recorded for nontargeted 5-FU/LPs was equal to (55.9 ± 6.6) and (42.9 ± 2.3)% when cells were treated at drug concentration of 2 µM for 48 and 96 h, respectively, thus highlighting their less significant cytotoxic efficacy respect to targeted anti-FZD10/5-FU/LPs.

For CaCo-2 cells, the bare 5-FU/LPs exhibited a time-dependent reduction of cell viability ranging from approximately 60% to 40% over the 24 to 96 h, for all tested concentrations (Figure 3B, grey bars). When the cells were incubated with free drug at the lowest tested concentrations (1 and 2 µM), the cell viability was only minimally affected at 24 and 48 h; conversely, a substantial reduction (*p* < 0.001 versus control) up to (38 ± 8)% in cell viability was recorded at 96 h (Figure 3B, blue bars). At the higher explored drug concentrations (3–10 µM), the free 5-FU exhibited a significant cytotoxic activity (*p* < 0.001 versus control) not only at 96, but also at 48 h (Figure 3B, blue bars). Cells exposure to anti-FZD10/5-FU/LPs induced a relevant cell-killing effect already within 24 h, throughout the drug concentrations range (Figure 3B, orange bars). Cell treatment with the engineered LPs significantly lowered (*p* < 0.001 versus control) the cell viability up to (40 ± 4) and (37 ± 6)% at 24 h, and up to (13 ± 2) and (16 ± 8)% at 96 h, at the tested drug concentrations equal to 1 and 2 µM, respectively. (Figure 3B, orange bars).

### 3.3. Effects of Formulated Liposomes on the Cell Morphology

**Figure 4:**
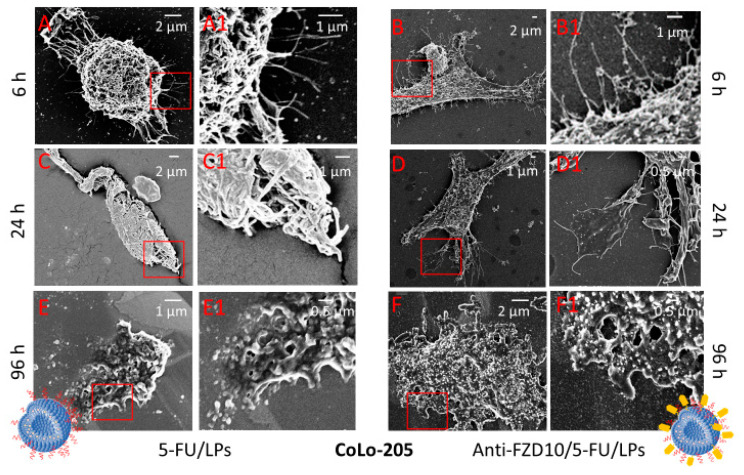
(FE-SEM) Representative field emission scanning electron microscopy micrographs(EHT) extra-high tension (EHT = 3.00 kV) and their corresponding close-up details at higher magnification of CoLo-205 cells after treatment with 5-FU/LPs (**A**,**A1**,**C**,**C1**,**E**,**E1**) and anti-FZD10/5-FU/LPs (**B**,**B1**,**D**,**D1**,**F**,**F1**) for 6, 24 and 96 h. 5-FU concentration: 2 µM.

**Figure 5:**
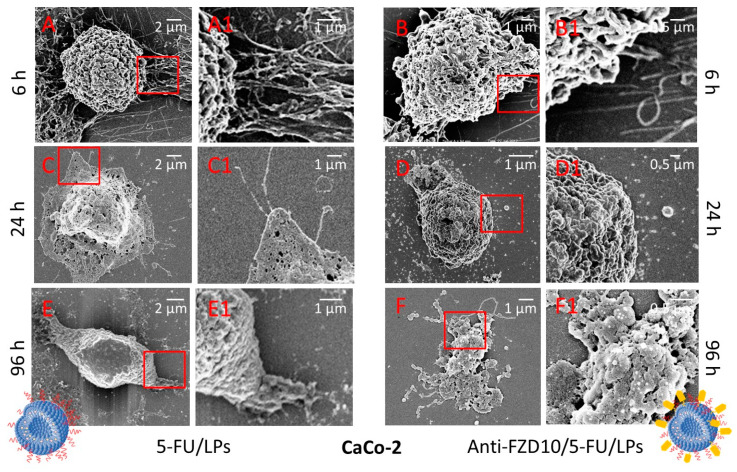
Representative FE-SEM micrographs (EHT = 3.00 kV) and their corresponding close-up details at higher magnification of CaCo-2 cells after treatment with 5-FU/LPs (**A**,**A1**,**C**,**C1**,**E**,**E1**) and anti-FZD10/5-FU/LPs (**B**,**B1**,**D**,**D1**,**F**,**F1**) for 6, 24 and 96 h. 5-FU concentration: 2 µM.

Morphological changes induced on CoLo-205 and CaCo-2 cells by exposure to anti-FZD10/5-FU/LPs and 5-FU/LPs (drug concentration of 2 µM) at 6, 24 and 96 h were observed at FE-SEM (Figure 4 and Figure 5).

In Figure S4 (Supplementary Materials), the pristine morphology of the untreated CaCo-2 and CoLo-205 cells was shown. The two cell lines appeared adherent, CaCo-2 cells are completely flat, while the CoLo-205 cells present a protruding spherical nucleus in the center of the flat cell body (Figure S4). FE-SEM analysis performed on both cell lines after treatment with 5-FU/LPs and anti-FZD10/5-FU/LPs for 6 h revealed that, the two liposomal formulations induced rounding of the cells, that acquired a spindle-shaped morphology as compared to the corresponding controls, with an increased number of pseudopodia due to the stress induced by incubation with 5-FU/LPs or anti-FZD10/5-FU/LPs (Figure 4A,A1,B,B1, and Figure 5A,A1,B,B1).

In the case of CoLo-205 cells, the 5-FU/LPs produced a more evident toxic effect compared to the anti-FZD10/5-FU/LPs after 24 h of incubation, revealing significant alterations in cell morphology and a strongly reduced number of pseudopodia (Figure 4C,C1,D,D1). Conversely, in the case of CaCo-2 cells, the anti-FZD10/5-FU/LPs mostly affected the cell morphology as compared to 5-FU/LPs. Indeed, CaCo-2 cell still resulted adherent to the substrate after treatment with the nontargeted liposomal formulations for 24 h (Figure 5C,C1,D,D1).

After 96 h exposure to both the liposomal formulations, CoLo-205 cells appear dead (Figure 4E,E1,F,F1), since no living cell structures were observed. The effectiveness of anti-FZD10/5-FU/LPs was also detectable on CaCo-2 cells at 96 h, since only cell fragments can be observed; conversely, a kind of morphology was still detected when CaCo-2 cells were treated with 5-FU/LPs for 96 h (Figure 5E,E1,F,F1).

### 3.4. Effectiveness of the Formulated Liposomes on Cell Migration

**Figure 6:**
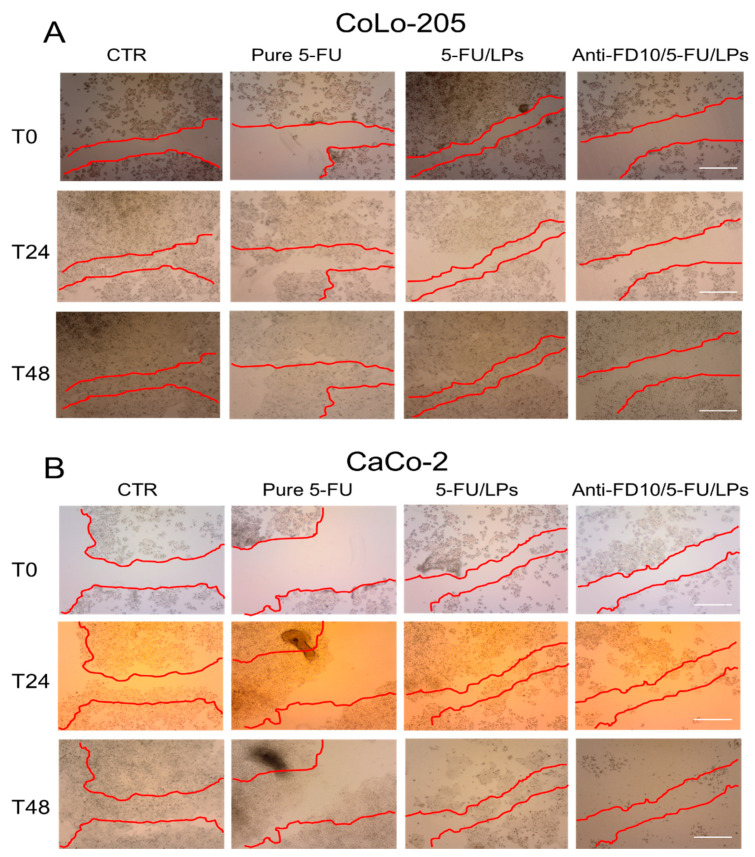
Qualitative analysis of collective CoLo-205 (**A**) and CaCo-2 (**B**) cell migration by in vitro scratch assay. Representative photomicrographs of scratch-wound closure of cells treated with free 5-FU, 5-FU/LPs or anti-FZD10/5-FU/LPs at different time points (0, 24 and 48 h). 5-FU concentration: 2 µM. CTR: untreated cells. Red lines represent the edges of the scratched areas. Scale bar: 200 µm.

The effect of the two formulated LPs on cell motility was assessed by performing the scratch assay on CoLo-205 and CaCo-2 cells. After creating the mechanical scratch (marked in red) on confluent cell monolayers, the cells were incubated with exogenous added free 5-FU, 5-FU/LPs or anti-FZD10 /5-FU/LPs (drug concentration 2 µM) and the effects were monitored at 0, 24 and 48 h (Figure 6A,B). CoLo-205 cells were able to wholly rescue the wound already within 24 h (Figure 6A), while for the nonmetastatic, untreated CaCo-2 cells, complete closure of the scratched area was observed within 48 h (Figure 6B). In the case of CoLo-205 cells, free 5-FU or 5-FU/LPs decreased cell migration after 24 h, although the widths of the injuries were still detectable. At 48 h, the scratched area appeared strongly reduced. Conversely, incubation with anti-FZD10/5-FU/LPs produced a substantial inhibition of cell migration in the wound regions; indeed, the scratched area was only partially reduced but still observable at both 24 and 48 h (Figure 6A). The results obtained by the scratch assay performed on CaCo-2 cells demonstrated a notable inhibition effect of 5-FU, free or encapsulated in the LPs, on cell migration (Figure 6B). Indeed, a considerable decrease of cell migration was observed, as compared with control cells, when the cells were exposed to the free 5-FU, although the area of injury appeared partially reduced at 24 and 48 h. In the case of 5-FU/LPs, CaCo-2 cells were able to partially migrate in the scratched area at 24 h, but the injury region was no longer recognizable at 48 h, since the cells appeared to be dead. The effect of anti-FZD10/5-FU/LPs on the migration and viability of CaCo-2 cells was more pronounced than that of nontargeted LPs; indeed, cell migration did not occur and death of the cells was already observed at 24 h. Remarkably, almost all the cells appeared to be dead at 48 h (Figure 6B).

### 3.5. Effectiveness of Formulated LPs on Vimentin and Phospho-Paxillin Cytoskeletal Proteins

**Figure 7:**
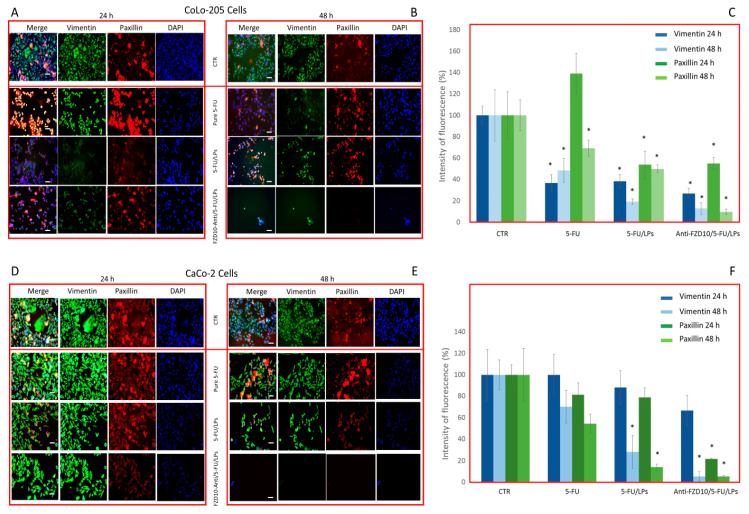
Detection and quantification of vimentin and phospho-paxillin (red) by immunofluorescence confocal microscopy in fixed CoLo-205 (**A**–**C**) and CaCo-2 cells (**D**–**F**), after cells incubation with free 5-FU, 5-FU/LPs or anti-FZD10/5-FU/LPs at 24 and 48 h. 5-FU concentration: 2 µM. CTR: untreated cells. Green channel: labeled vimentin, red channel: labeled phospho-paxillin, blue channel: labeled nuclei (DAPI), and corresponding overlay (Merge). Scale bar: 50 µm. (*) *p* < 0.001 versus control (CRT).

The expression level of two specific proteins, namely vimentin and phospho-paxillin, involved in cell stability and cell adhesion, respectively, was assessed by immunofluorescence imaging in both fixed CoLo-204 and CaCo-2 cells, after incubation with 5-FU, 5-FU/LPs or anti-FZD10/5-FU/LPs at 24 and 48 h. The tested 5-FU concentration was 2 µM (Figure 7). The confocal microscopy investigation performed on CoLo-205 cells treated with free 5-FU (Figure 7A–C) indicated a significant, time-dependent reduction of vimentin expression (green labeled), being (36.8 ± 7.5)% and (48.6 ± 11.2)% the percentage ratio (compared to the control) of mean fluorescence intensity for the cells treated at 24 and 48 h, respectively (*p* < 0.001 versus control). Conversely, in the case of phosphor-paxillin (red labeled), the percentage ratio of fluorescence intensity was firstly increased, at 24 h ((139.1 ± 19.3)% and then reduced to (69.3 ± 7.4)% at 48 h (*p* < 0.001 versus control).

The confocal images of CoLo-205 cells after treatment with 5-FU/LPs or anti-FZD10/5-FU/LPs show that a significant reduction (*p* < 0.001 versus control) of the expression level of each investigated protein was always observed at both cell incubation times. Indeed, in the case of vimentin, the percentage ratios of mean fluorescence intensity were equal to (38.4 ± 6.3)% and (26.9 ± 4.9)% at 24 h and (19.3 ± 2.4)% and (12.8 ± 5.5)% at 48 h, for cells treated with 5-FU/LPs or anti-FZD10/5-FU/LPs, respectively; in the case of phospho-paxillin, the corresponding percentage ratios were (53.9 ± 12.7)% and (55.0 ± 5.5)% at 24 h and (49.9 ± 3.4)% and (9.7 ± 2.5)% at 48 h (Figure 7A–C). The immunofluorescence analysis carried out by confocal microscopy on CaCo-2 cells treated with free 5-FU (Figure 7D–F) demonstrated that although the expression levels of vimentin and phospho-paxillin decreased in a time-dependent manner, the observed decreases of fluorescent signals were not significant. On the contrary, a significant decrease (*p* < 0.001 versus control) in the expression levels of vimentin and phospho-paxillin was observed when CaCo-2 cells were incubated with 5-FU/LPs for 48 h. The percentage ratio of mean fluorescence intensity was found to be (28.2 ± 15.2)% and (14.0 ± 3.5)% for vimentin and phospho-paxillin, respectively (Figure 7D–F). A significant (*p* < 0.001 versus control), detectable decrease in the expression level of phospho-paxillin was observed in CaCo-2 cells treated with anti-FZD10/5-FU/LPs for 24 and 48 h (percentage ratio of mean fluorescence intensity (21.7 ± 0.5)% and (5.3 ± 0.7)% at 24 and 48 h, respectively), while in the case of vimentin, a significant difference in the expression level, compared to the control (*p* < 0.001 versus control), was recorded only at 48 h (percentage ratio of mean fluorescence intensity (5.2 ± 4.6)%, (Figure 7D–F)). For both cell lines, the reductions in fluorescence intensity observed for the blue-stained cell nuclei, compared to the corresponding control cells, when the cells were treated with the two formulated LPs or free 5-FU at 24 and 48 h, were in agreement with the results obtained at the cell viability assay (Figure 3).

## 4. Discussion

Currently, 5-FU is one of the first-line chemotherapeutic agents for the treatment of advanced CRC. It is a typical antimetabolite with a strong time-dependent mode of action [27]. Despite its therapeutic efficacy, 5-FU has some limitations, mainly tumor cell resistance and a short biological half-life. Consequently, multiple administrations of high doses, of which only a very low percentage reaches the tumor area, are required, and severe systemic (gastrointestinal, hematological, cardiac, and dermatological) toxicities occur. Indeed, 5-FU has been demonstrated to induce cardiotoxicity, followed by thrombosis, and alterations of the antioxidant defense capacities in myocardial tissues, with increases in the cardiac enzymes superoxide dismutase and glutathione peroxidase [28,29]. Therefore, the development of new delivery methods that selectively target the abnormal colonic mucosa could offer several benefits to patients affected by advanced CRC [30,31,32]. In this perspective, several nanovectors, such as lipid-based nanoformulations, nanogels, polymer-based micro/nanoparticles, carbon nanotubes and polysaccharides-based nanosystems have been designed to achieve an effective delivery of cytotoxic drugs and a selective targeting of CRC sites [33,34] Among them, targeted LPs have been demonstrated to exhibit an enhanced antitumor activity on CRC cells as compared to nontargeted counterparts. Folate, mannose and integrin receptors, overexpressed on CRC cells, are the main targets that have been explored for the development of CRC-targeted LPs [33]. Specifically for 5-FU, immune-LPs conjugated with proper folate or integrinβ6 antibodies have been proposed for CRC treatment [11,35,36]. Furthermore, O. Udofot et al. reported a study of the cytotoxic effect of 5-FU loaded pH-sensitive LPs on CRC cell lines; in this case, the LPs were conjugated with a selected anti-EGFR antibody [37]. In this study, PEG-functionalized 5-FU loaded LPs were covalently conjugated with a specific antibody able to recognize and bind the FZD10 surface cell receptor, to create innovative immuno-LPs that could potentially be useful for targeted therapy of CRC. In particular, FZD10 targeted and nontargeted LPs were prepared and characterized by complementary optical and morphological techniques. The average hydrodynamic diameter values obtained by DLS resulted lower than 200 nm for both formulated LPs. It is well known in literature that LPs of sizes between 100 and 300 mn are able to extravasate and localize in the tumor tissue, exploiting the EPR effect [38]. ζ-potential measurements proved that LPs exhibited a high negative surface charge, that further increased after LPs conjugation with FZD10 antibody, together with values of less than−30 mV for both formulated LPs, thus indicating their high colloidal stability in aqueous media and suggesting a good cell internalization capacity of the vesicles [39]. The encapsulation efficiency values achieved for 5-FU/LPs and anti-FZD10/5-FU/LPs are in agreement with the data already reported in the literature concerning other 5-FU encapsulating LPs [40].

The in vitro studies were performed on two selected CRC cell lines, namely CaCo-2 and CoLo-205. Preliminarily, quantitative and qualitative evaluation of the cellular FZD10 expression in CaCo-2 and CoLo-205 cells was made by immunoblotting and immunofluorescence, respectively, to validate the use of the FZD10 engineered LPs for targeted treatment of CRC (see Supplementary Materials). Furthermore, previous data had demonstrated the abundance of expression of FZD10 protein on CRC tissues, especially at late stages and in metastatic tissues [18]. Cell viability evaluation was conducted by testing the drug concentrations in the range of clinically relevant concentrations of 5-FU, previously reported in the literature and identified by performing clinical studies on pharmacokinetic modulating chemotherapy. E, Ojima et al. reported the range between 0.1 and 10 µM, while Beumer, J.H. et al. the range between 0.6 and 13 µM [41,42]. The two ranges slightly differ from each other, owing to different techniques used for the drug detection, modality of exposure to drug and substantial interindividual pharmacokinetic variability [42,43]. The results indicated that the cytotoxic activity of 5-FU is significantly enhanced when delivered by anti-FZD10/5-FU/LPs at the lowest tested drug concentrations (1 and 2 µM), as compared not only to the free drug but also to the nontargeted LPs. Therefore, at the lowest tested drug concentrations, the data obtained suggest that a more controlled drug release and higher cell accumulation of the targeted LPs occurred, likely due to their improved cell-internalization ability, exploiting not only passive transport but also antibody-receptor binding, allowing the use of lower 5-FU concentrations, and achieving a higher expected performance in terms of reduced toxicity effects. Therefore, the 5-FU concentration of 2 µM was further considered for the in vitro studies. 

The response induced on cell morphology after cell treatment with the two formulated LPs was investigated by means of the FE-SEM technique, that produces detailed images of a whole cell and ensures the visualization of several morphological details of a single cell (namely size, shape, nuclear/cytoplasmic ratio, number of pseudopodia and cell adherence), thus enabling any cell morphological changes induced by cell treatment with a specific pharmacological agent to be evaluated [27]. FE-SEM investigation visualized the several, severe time-dependent effects induced on the morphology and consequent viability of CaCo-2 and CoLo-205 cells, after treatment with 5-FU/LPs or anti-FZD10/5-FU/LPs. CoLo-205 cells appeared dead at 96 h treatment with both the formulated LPs, while, in the case of CaCo-2 cells, the anti-FZD10/5-FU/LPs exhibited a more efficacious cytotoxic activity than the 5-FU/LPs at 96 h, thus corroborating the data shown by MTS assay.

We also investigated the effect of the two formulated LPs on cell migration, a key factor in the malignant spread of cancer. For the metastatic CoLo-205 cells, the anti-FZD10/5-FU/LPs were found to induce a more significant inhibition of cell migration in the test area than the free 5-FU and 5-FU/LPs, the wound regions being only partially reduced at both 24 and, remarkably, at 48 h. The inhibition effect on migration of nonmetastatic CaCo-2 cells after treatment with 5-FU, free or embedded in the LPs, resulted more evident compared to CoLo-205 cells. Once again, the anti-FZD10/5-FU/LPs more consistently inhibited the cell migration compared to the free 5-FU and nontargeted LPs at 24 and 48 h treatment. Furthermore, the death of most of the cells was observed at 48 h.

Immunofluorescence analysis, performed on both fixed cell lines after incubation with free 5-FU, 5-FU/LPs or FZD10-anti/5-FU/LPs, was used to monitor the expression level of two proteins, namely vimentin and phospho-paxillin. The role of vimentin and phospho-paxillin as active players in cell focal adhesion and migration, as well as in pathological conditions, including cancer development and metastasis, is widely discussed in literature. The two proteins are found to be overexpressed in several cancer tissues and metastatic cancer cells, including also CRC, especially during adhesion and distant metastases formation [44,45,46]. High expression levels of both the vimentin and paxillin have been found to significantly contribute both to the malignancy and drug-resistance of CRC [47,48,49]. Consequently, the downregulation of the expression levels of the two proteins was found to be related to a reduction of metastatic development [50,51,52,53]. The investigation proved the time-dependent reduction in the fluorescence intensity of the vimentin and phospho-paxillin, confirming the effective activity of 5-FU on the inhibition of CRC cell proliferation [54]. The immunofluorescence analysis also indicated an enhanced toxic effect of 5-FU, when incorporated in the formulated LPs. In particular, the anti-FZD10/5-FU/LPs more consistently lowered the expression of the two proteins at 48 h in the case of CoLo-205, and already at 24 h treatment in CaCo-2 cells. 

The overall results suggest that, at the lowest tested drug concentrations, the cytotoxic activity of targeted LPs was enhanced, as compared to free 5-Fu and nontargeted LPs. Although further studies are planned to investigate the specific mechanisms involved in the cell uptake, our preliminary findings are encouraging to validate the FZD10 protein as a novel, effective target for CRC. We expect that in future clinical applications, the use of anti-FZD10/5-FU/LPs could allow a reduction of the 5-FU doses administered, while maximizing the therapeutic efficacy. A new, promising scenario for targeted CRC therapy may be envisaged: administration of the anti-FZD10/5-FU/LPs here proposed, encapsulated in softgel capsules or suppositories, could provide an effective route for oral or rectal delivery, as employed for other compounds that need to reach the digestive canal, resisting gastric acids [55]

## Figures and Tables

**Table 1:** Intensity-average hydrodynamic diameter and corresponding polydispersity index (PDI) determined by Dynamic Light Scattering (DLS), ζ-potential, and encapsulation efficiency (EE%) of 5-FU/LPs and anti-FZD10/5-FU/LPs.

Samples	D_h_ (nm)	PDI	ζ-Potential (mV)	Drug EE (%)
**5-FU/LPs**	155 ± 47	0.32 ± 0.05	−37.73 ± 5.38	65.9 ± 2.9
**Anti-FZD10/5FU/LPs**	193 ± 12	0.33 ± 0.15	−43.43 ± 4.75	45.7 ± 5.9

## Supplementary Materials

The following are available online at https://www.mdpi.com/1999-4923/12/7/650/s1, Figure S1. UV-Vis absorption spectrum of FZD10-anti/5-FU/LPs before (A) and after (B) incubation with Alexa Fluor 555-conjugated secondary antibody. Figure S2. In vitro release of 5-FU from anti-FZD10/5-FU/LPs as a function of time, at 37 °C. Data are reported as mean value (±standard deviation) calculated on three replicates. Figure S3. (A) Representative Western blotting of FZD10 and GAPDH housekeeping protein and (A1) semi-quantitative estimation, by densitometry analysis of protein bands, of relative FZD10 expression levels in untreated HCEC-1CT, CaCo-2 and CoLo-205 cells, after loading the same total protein content (20 µg). For the semiquantitative analysis, FZD10 bands are evaluated after normalization with the corresponding housekeeping GAPDH protein band, for each sample. (*) *p* < 0.001 versus negative control (HCEC-1CT cells). (B) Detection of FZD10 by immunofluorescence confocal microscopy in fixed HCEC-1CT, CaCo-2 and CoLo-205 cells. Blue channel: nuclei; green or red channel: labeled FZD10 and corresponding overlay. Scale bar 50 µm. Figure S4. Representative FE-SEM micrographs (EHT = 3.00 kV) of untreated CoLo-205 (A) and CaCo-2 (B) cells.
