# Supplementary material for: Effectiveness of a Controlled 5-FU Delivery Based on FZD10 Antibody-Conjugated Liposomes in Colorectal Cancer In vitro Models
Source: Pharmaceutics. 2020 Jul 10;12(7):650. doi: 10.3390/pharmaceutics12070650 (PMC7408534; doi:10.3390/pharmaceutics12070650)
Supplement: Supplementary file 1 [file pharmaceutics-12-00650-s001.pdf]

pharmaceutics

MDPI

Supplementary Materials: Effectiveness of a Controlled 5-FU Delivery Based on FZD10 Antibody-Conjugated Liposomes in Colorectal Cancer In vitro Models.

Maria Principia Scavo, Annalisa Cutrignelli, Nicoletta Depalo, Elisabetta Fanizza, Valentino Laquintana, Giampietro Gasparini, Gianluigi Giannelli and Nunzio Denora

Indirect detection of FZD10 antibody conjugated with liposomes surface

Lyophilized FZD10-anti/5-FU/LPs were reconstituted in 300 μL of PBS (pH 7.5) and incubated with with 5 μg of Alexa Fluor 555 conjugated secondary antibody (goat anti-Rabbit IgG (H + L) cross-adsorbed secondary antibody, Alexa Fluor 555) for one hour at room temperature. Then, the labelled anti-FZD10/5-FU/LPs were recovered as pellet by ultracentrifugation of the reaction mixture at 10000 × g at 4 °C for 40 min to remove the excess of the secondary antibody and finally characterized by UV-Vis spectroscopy analysis (UV/vis/NIR Cary 5 spectrophotometer, Varian).

The indirect detection of FZ10-antibody on the LPs surface was achieved by using UV-Vis spectroscopy analysis. In Figure S1B, the absorption spectrum of anti-FZD10/5-FU/LPs after incubation with labelled secondary antibody clearly shows the presence of a peak centered at 555 nm, due to the binding of the dye (Alexa 555) conjugated secondary antibody to the surface of anti-FZD10/5-FU/LPs. Conversely, no peak can be observed in the absorbance spectrum of untreated anti-FZD10/5-FU/LPs (Figure S1A), thus evidencing the successful of the conjugation process.

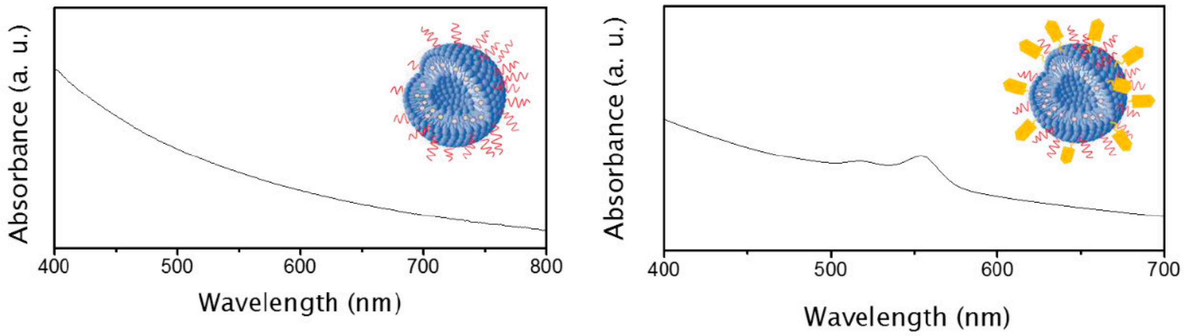

Figure S1. UV-Vis absorption spectrum of anti-FZD10/5-FU/LPs before (**A**) and after (**B**) incubation with Alexa Fluor 555 conjugated secondary antibody.

In vitro drug release

In Figure S2, was reported the drug release study for anti-FZD10 /5-FU/LPs, monitored by UV-Vis absorption spectroscopy. A burst in the 5-FU release up to of 8% was observed over the first 3 h, as consequence of desorption of 5-FU from surface of anti-FZD10/5-FU/LPs and its diffusion into the outer medium. Subsequently, a sustained release of 5-FU up to 17% from the anti-FZD10/5-FU/LPs was observed within 48 h.

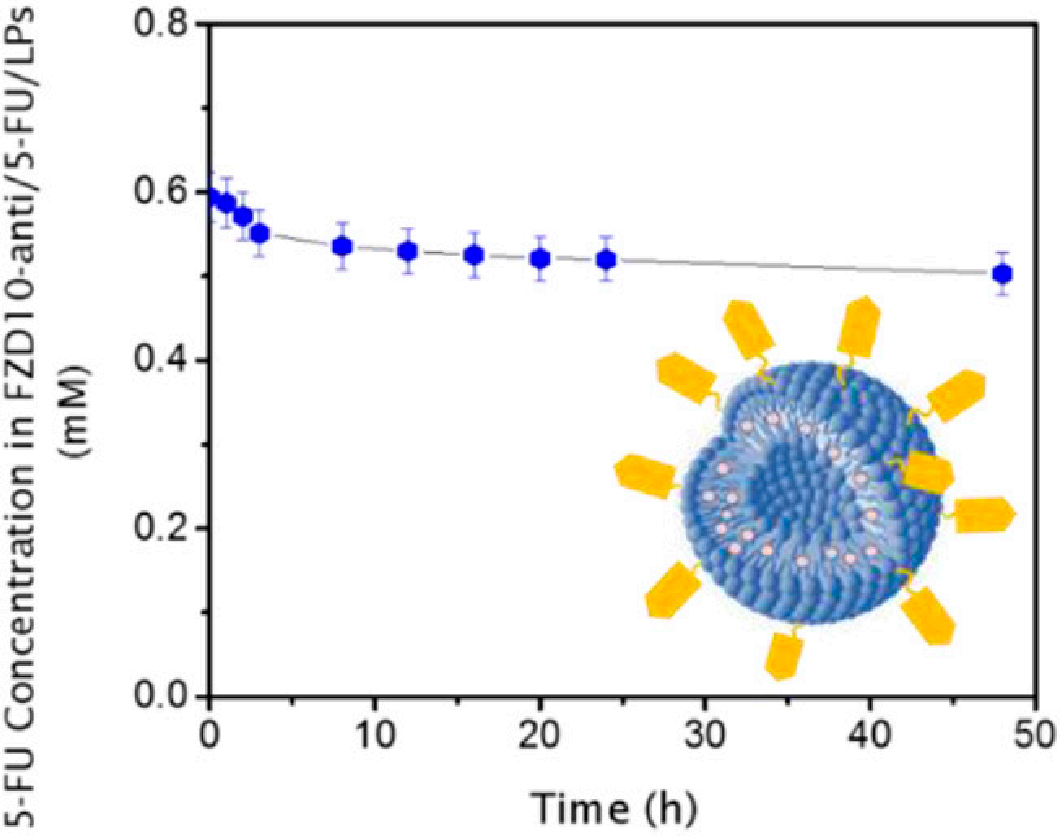

Figure S2. In vitro release of 5-FU from anti-FZD10/5-FU/LPs as function of the time, at 37 °C. Data are reported as a mean value (± standard deviation) calculated on three replicates.

FZD10 immunodetection on CaCo-2 and CoLo-205 cells

To define the expression of FZD10 on CaCo-2 and CoLo-205 cells, immunofluorescence and Western blotting experiments were performed. Normal epithelial cell line, namely HCEC-1CT, was cultured into Colo-Up (HCEC-1CT Growh Medium ready to use from Evercyte), added with 1% of penicillin/streptomycin. When the cell confluence was about 50%, the cell layer was rinsed with PBS, trypsinized, and consequently subculturing for the in vitro experiments of Western blotting and immunofluorescence. Briefly, for immunofluorescence investigation, each cell line was seeded, namely HCEC-1CT, CaCo-2 and CoLo-205, at a density of 10×10^3 into four-well slides chambers, and when the semiconfluence was reached, the cells were rinsed three times with PBS and fixed with 96% cold ethanol for 15 min. Subsequently, the cells were first washed three times with PBS, then permeabilized with 0.5% Tritox for 10 min and finally nonspecific sites were blocked with 5% BSA. The primary antibody, rabbit polyclonal anti human anti-FZD10 (AbCam), was diluted in the blocking solution, and the samples were incubated at 4 °C overnight. Then, the cells were washed twice with PBS and incubated for one hour at room temperature in a dark side with a specific green-fluorescent conjugated secondary IgG Alexa 555 anti rabbit (red), for HCEC-1CT, CaCo-2 or IgG Alexa 488 anti rabbit (green) for CoLo-205 (Thermo Fisher Scientific, Waltham, MA, USA). After washing with PBS, the cells were mounted using a prolonged gold antifade reagent containing DAPI (blue).

For Western blotting analysis, HCEC-1CT, CaCo-2 and CoLo-205 cells, were lysated by using 1X radio immunoprecipitation buffer (RIPA; Cell Signaling Technology, Danvers, MA, USA) containing protease inhibitors (Amresco, Solon, OH, USA), and the total proteins contained in the lysate was measured by means of Bradford kit assay (Bio-Rad Hercules, CA, USA). 20 μg of total extracted proteins from the cells of each lines samples, was mixed with reducing Laemmli-buffer, loaded on 4–15% Tris-glycine sodium dodecyl sulfate- polyacrylamide gels (Bio-Rad, Hercules, CA, USA) and

electrophoresed. Subsequently, proteins were blotted to PVDF membranes (Bio-Rad, Hercules, CA, USA) using Trans-blot system (Bio-Rad, Hercules, CA, USA). The blotted membranes were treated with 5% non-fat milk (Bio-Rad, Hercules, CA, USA) in Tris-buffered saline-supplemented with 0.05% Tween-20 (TBS-T) for one hour, to block nonspecific binding sites, they then were incubated with primary antibodies, namely anti-FZD10 (1:500, Abcam, Cambridge, UK), anti-GAPDH (1:1000, Abcam, Cambridge, UK). After three washings with TBS-T, membranes were incubated with corresponding HRP-conjugated secondary antibodies (1:1000, Santa Cruz, CA, USA) for one hour at room temperature and subsequently washed with TBS-T. The chemiluminescence signals from proteins were imaged after incubation by using an enhanced chemiluminescence kit (Bio-Rad, Hercules, CA, USA) by Chemidoc XRS + (Bio-Rad, Hercules, CA, USA). The images were analyzed by using Image Lab 5.2.1 software.

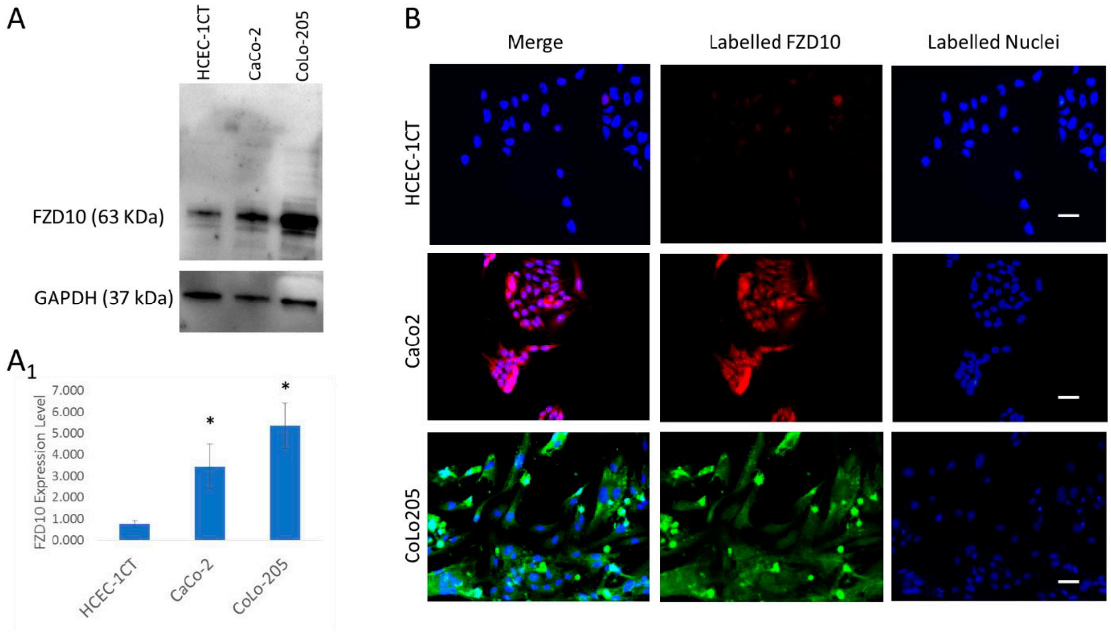

Figure S3. (**A**) Representative Western blotting of FZD10 and GAPDH housekeeping protein and (**A1**) semiquantitative estimation, by densitometry analysis of protein bands, of relative FZD10 expression level in untreated HCEC-1CT, CaCo-2 and CoLo-205 cells, by loading the same total protein content (20 µg). For the semiquantitative analysis, FZD10 bands are evaluated upon normalization with the corresponding housekeeping GAPDH protein band, for each sample. (*) $p < 0.001$ versus negative control (HCEC-1CT cells). (**B**) Detection of FZD10 by immunofluorescence confocal microscopy in fixed HCEC-1CT, CaCo-2 and CoLo-205 cells. Blue channel: nuclei; green or red channel: labeled FZD10 and corresponding overlay. Scale bar 50 µm.

The cellular FZD10 expression in CaCo-2 and CoLo-205 cells was quantitatively evaluated by immunoblotting and qualitatively detected by immunofluorescence confocal microscopy, to validate the use of FZD10 functionalized LPs for the targeted treatment of CRC. Normal epithelial cell line, was used as negative control. Western Blotting analysis carried out on untreated HCEC-1CT, CaCo-2 and d CoLo-205 cells, confirmed the presence of the FZD10 in the all tested cells (Figure S3A). The semiquantitative analysis of the average FZD10 expression level in the cell extracted proteins samples, normalized by using the corresponding housekeeping GAPDH bands (Figure S3A1), proved that FZD10 expression was significantly higher in CaCo2 and Colo205 cells if compared to normal cells (HCEC-1CT) used as negative control ($p < 0.001$). In figure S3B the cellular immunofluorescence images show a low fluorescence intensity of labelled FZD in normal cells if compared to both cancer cell lines where the protein expression was revealed on the cellular membrane and into cytoplasm, in particular in CoLo-205 cells.

SEM investigation on untreated CaCo-2 and CoLo-205 cells

In Figure S4, representative FE-SEM micrographs of untreated CoLo-205 (A) and CaCo-2 (B) cells are reported.

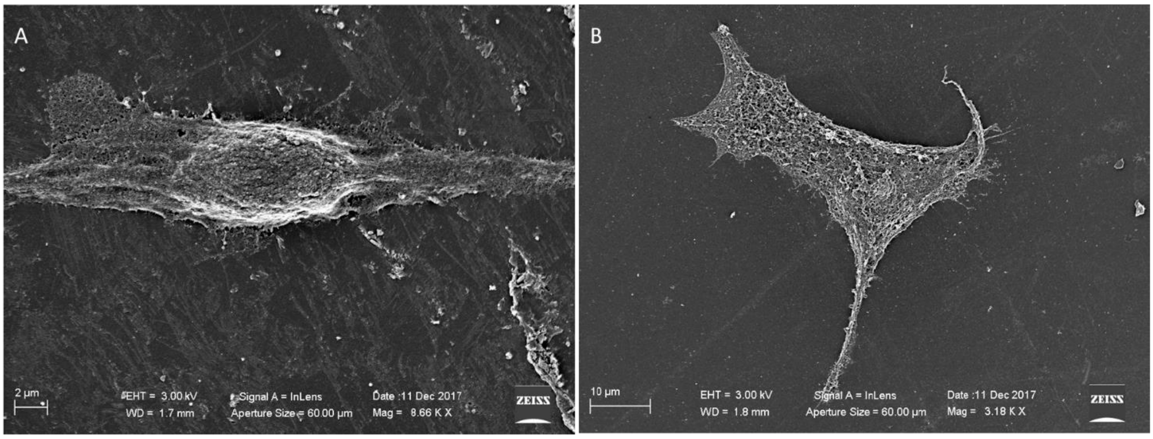

Figure S4. Representative FE-SEM micrographs (EHT = 3.00 kV) of untreated CoLo-205 (**A**) and CaCo-2 (**B**) cells.
